# Shape engineering vs organic modification of inorganic nanoparticles as a tool for enhancing cellular internalization

**DOI:** 10.1186/1556-276X-7-358

**Published:** 2012-07-01

**Authors:** Didem Sen Karaman, Diti Desai, Rajendran Senthilkumar, Emma M Johansson, Natalie Råtts, Magnus Odén, John E Eriksson, Cecilia Sahlgren, Diana M Toivola, Jessica M Rosenholm

**Affiliations:** 1 Centre for Functional Materials, Laboratory of Physical Chemistry, Department of Natural Sciences, Åbo Akademi University, Porthansgatan 3-5, Turku, FI-20500, Finland; 2 Pharmacy Department, Faculty of Tech. & Eng, The M.S. University of Baroda, Vadodara, Gujarat -390002, India; 3 Department of Biosciences, Cell biology, Åbo Akademi University, Artillerigatan 6A, Turku FI-20520, Finland; 4 Nanostructured Materials Division, Department of Physics, Chemistry and Biology, Linköping University, Linköping, SE-581 83, Sweden; 5 Turku Centre for Biotechnology, University of Turku and Åbo Akademi University, P.O. Box 123, Turku FI-20521, Finland; 6 Turku Center for Disease Modeling, Kiinamyllynkatu 10, Turku FIN-20520, Finland

**Keywords:** mesoporous silica nanoparticles, surface functionalization, rod-shaped particles, surface charge, cellular internalization, nanomedicine

## Abstract

In nanomedicine, physicochemical properties of the nanocarrier affect the nanoparticle's pharmacokinetics and biodistribution, which are also decisive for the passive targeting and nonspecific cellular uptake of nanoparticles. Size and surface charge are, consequently, two main determining factors in nanomedicine applications. Another important parameter which has received much less attention is the morphology (shape) of the nanocarrier. In order to investigate the morphology effect on the extent of cellular internalization, two similarly sized but differently shaped rod-like and spherical mesoporous silica nanoparticles were synthesized, characterized and functionalized to yield different surface charges. The uptake in two different cancer cell lines was investigated as a function of particle shape, coating (organic modification), surface charge and dose. According to the presented results, particle morphology is a decisive property regardless of both the different surface charges and doses tested, whereby rod-like particles internalized more efficiently in both cell lines. At lower doses whereby the shape-induced advantage is less dominant, charge-induced effects can, however, be used to fine-tune the cellular uptake as a prospective ‘secondary’ uptake regulator for tight dose control in nanoparticle-based drug formulations.

## Background

Whereas nanotechnology seeks to engineer and apply the unique properties of materials that emerge when the dimensions enter the nanoscale, nanomedicine attempts to exploit these for the benefit of human health. Nanomaterials in this context are intriguing because they can resemble biological ‘nanomachines’ (such as biomacromolecules) as they meet on the same length scale [1] and, thus, can be expected to perform similar tasks or at least possess reminiscent biobehavior. One of the properties exploited in nanomedicine is the ability of nanoparticles to enter cells, whereby they can function as efficient carriers for intracellular drug delivery. The behavior of nanomaterials on the nano-bio interface is largely determined by the physicochemical properties of the nanocarrier, of which size and surface charge have been emphasized as the two most critical ones. Another design parameter that has not received as much attention to date is particle shape [2]. Available results suggest that, as compared to their spherical counterparts, elongated nanoparticles could be more favorable for therapeutic applications based on targeting specificity, biodistribution as well as cellular internalization profiles [3]. On this topic, it has been suggested that the resemblance of rod-like particles with rod-like bacteria could be a reason for the observed advantages in internalization rates in non-phagocytic cells [4]. Particles that, in such a way, mimic properties such as size, shape and flexibility of naturally occurring entities (for instance, circulating cells, e.g., red blood cells) may offer advantages that are typically not observed for standard (polymeric) particles [5].

One reason for the general scarcity of the shape-dependency effects on biological performance is the more difficult fabrication of nanoparticles with controlled rod-like morphology due to surface energy minimization during synthesis, leading to spherical shapes [6]. Especially for more novel materials' classes, the first hurdles to overcome include obtaining well-dispersed, monosized particles throughout all processing steps [7]. One promising materials' class in the sense of nanomedicine is mesoporous silica, which can be produced with nanoscale particle diameters and with tunable properties when it comes to both particle size and morphology, meso (pore) structure and size as well as surface chemistry [8]. The controllable characteristics of these materials have boosted research regarding their potential use within the biomedical field during the last decade, especially so for targeted cancer therapy and diagnostics [9-11]. Mesoporous silica nanoparticles (MSNs) have been successfully loaded with a range of different chemotherapeutics for efficient intracellular delivery and subsequent elimination of the cancer cells. A vast array of sizes and surface modifications have been produced and studied both *in vitro* and *in vivo*, while only a few studies have been devoted to the shape effect on MSN biobehavior [12-16]. To add to the increased understanding of shape-induced effects for MSNs, we have, in this study, synthesized two similarly sized but differently shaped rod-like (NR-MSP) and spherical (S-MSP) fluorescently labeled mesoporous silica nanoparticles in order to investigate the morphology effect on cellular internalization. Additionally, both the rods and spheres were functionalized to yield different surface charges in order to distinguish morphology from surface charge-induced effects and, in the best case scenario, find the dominant parameter. *In vitro* studies were carried out in two different cancer cell lines, HeLa (cervical carcinoma cells) and Caco-2 (human epithelial colorectal adenocarcinoma cells), to investigate the particle characteristic impact on cells of different origin. According to our results, all of the studied factors (particle shape, surface charge, the nature of the coating as well as the targeted cell populations) are important to be considered when designing new nanocarrier formulations for targeted cancer therapies or other potent drugs that require tight dose control.

## Methods

### Particle preparation and characterization

**Figure 1:**
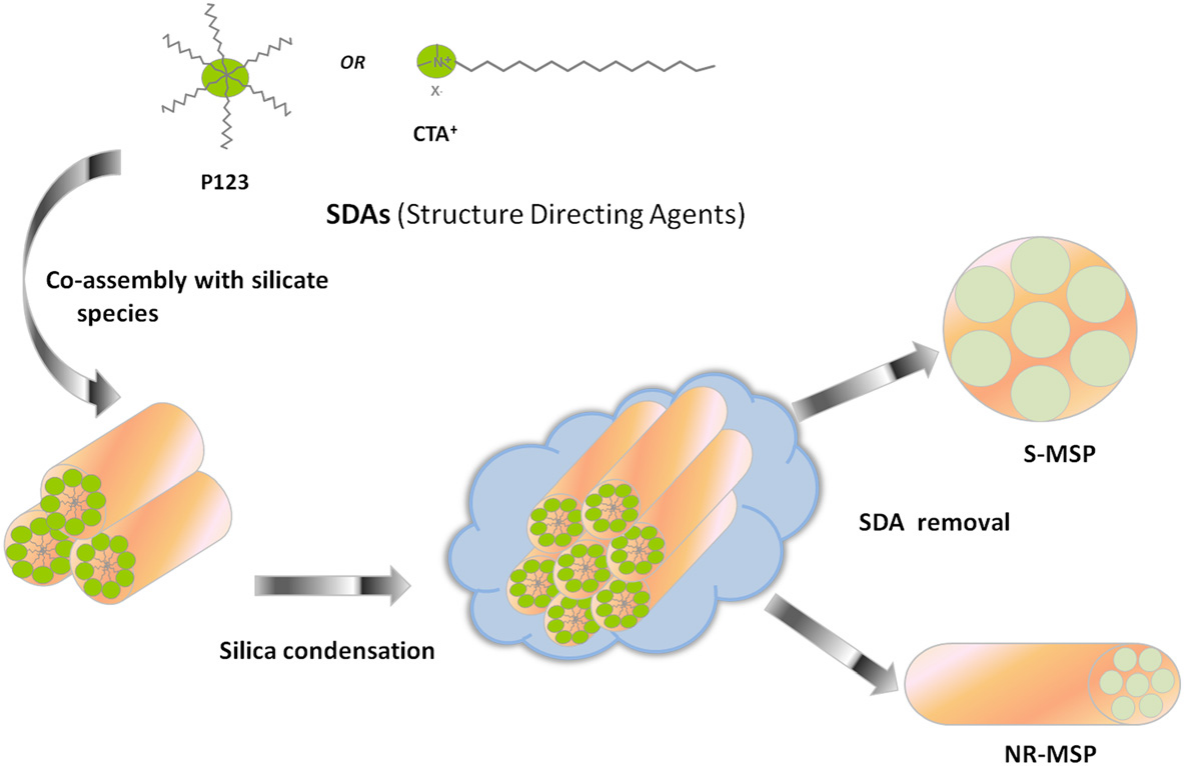
Brief summary of the formation of spherical and rod-like mesoporous silica nanoparticles (denoted MSP).

**Figure 2:**
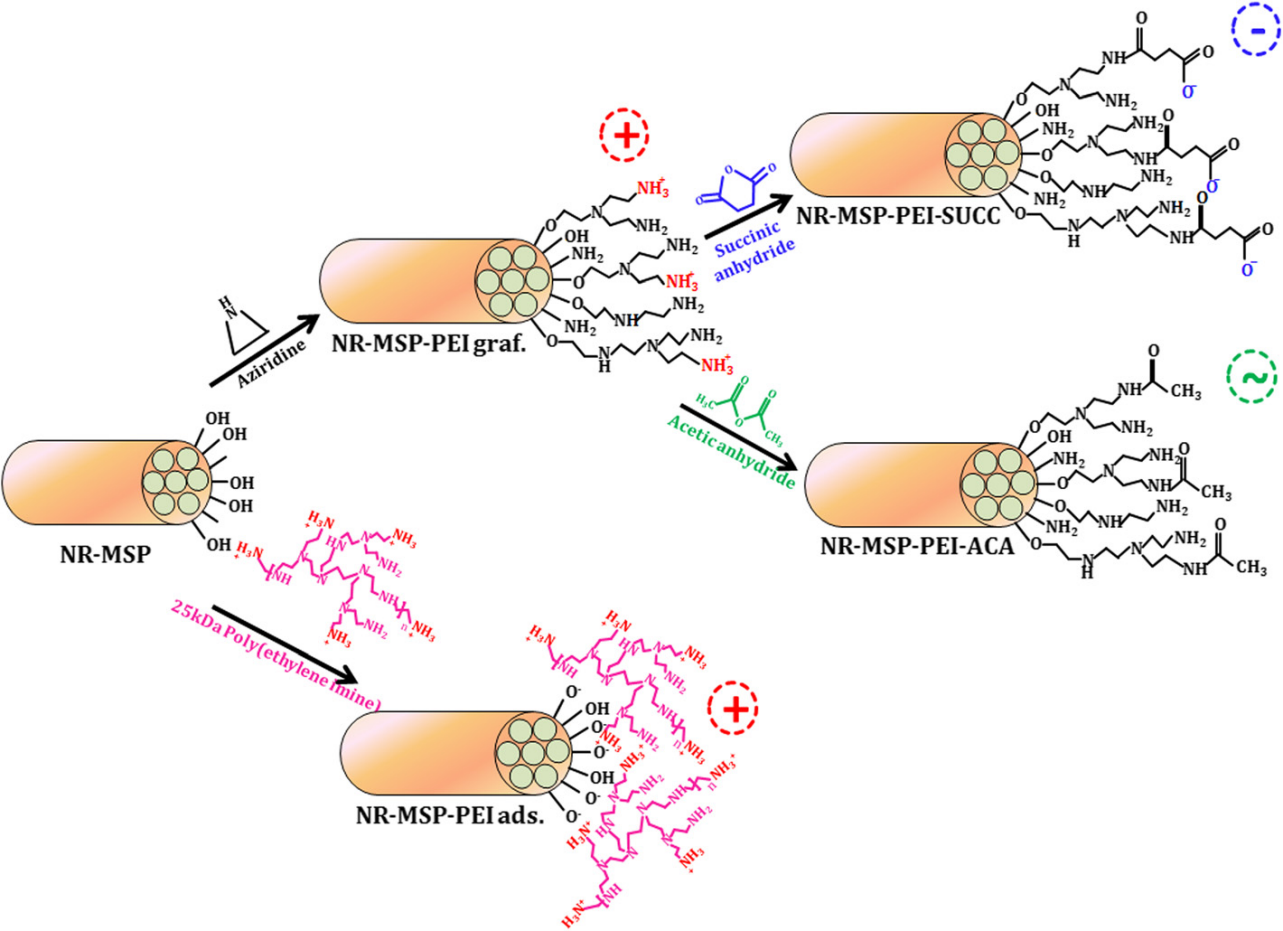
The applied surface functionalization procedures for spherical and rod-shaped mesoporous silica nanoparticles.

The synthesis and functionalization of spherical and rod-like MSNs have been summarized below in Figures 1 and 2, respectively.

### Spherical mesoporous silica particles

S-MSP_1_ was synthesized according to the protocol described in reference [17], with slight modifications and details given in Additional file 1. In order not to alter the surface charge of the particles, fluorescein isothiocyanate (FITC)-modified aminopropyltriethoxysilane silane (APTES) was mixed with the silica source before adding to the reaction solution to provide co-condensed functionalization of FITC within the silica framework. The modification of APTES was carried out by pre-reacting FITC with APTES in 2 mL acetone with a molar ratio of 1:3 and stirring for 2 h under inert atmosphere. The molar ratio between APTES and tetraethyl orthosilicate (TEOS) was kept as 1:100. The thus-preserved negative surface charge was subsequently utilized for further electrostatic adsorption of branched 25 k poly(ethylene imine) (PEI) to the surfactant-extracted particles (S-MSP_1_-PEI adsorbed). The functionalization of S-MSP_1_ was carried out by overnight adsorption (Figure 2) in 4-(2-hydroxyethyl)-1-piperazineethanesulfonic acid (HEPES) buffer at neutral pH, after which, the particles were collected by centrifugation and washed excessively with deionized water to remove excess PEI.

S-MSP_2_ was synthesized according to our previously published protocols [18], with FITC added in the synthesis step to create inherently fluorescent particles. In this case, no pre-reaction between the aminosilane and FITC was needed as the amount of aminosilane used in the synthesis was considerable (10 mol%), and the reaction conditions are favorable also for FITC conjugation. As the surface charge of the MSPs prepared according to this protocol is neutral to slightly positive, electrostatic adsorption of PEI is not possible. Thus, these particles were PEI-functionalized by surface growing of PEI [19-21] to yield sample S-MSP_2_-PEI grafted (Figure 2).

### Rod-like mesoporous silica particles

NR-MSPs were synthesized according to reference [22], with TEOS as silica source and block-co-polymer P123 (EO_20_PO_70_EO_20_) as pore-structuring agent in the presence of NH_4_F and heptane. Contrary to the S-MSP syntheses, the nanorod (NR) synthesis is performed under acidic conditions, where the particle length and width can be tuned with the aid of HCl concentration. The synthesis solution consisted of a molar ratio of 1 P123/1.8 NH_4_F/280 Heptane/60 TEOS/356 HCl/10335 H_2_O. The synthesis was kept under vigorous stirring for 4 min and subsequently, under static conditions for 1 h. Then, the solution was transferred to a closed Teflon flask for hydrothermal treatment at 100°C for 24 h. The resulting product was filtered and washed with distilled water. Finally, the material was calcined at 550°C for 5 h in order to remove the structure-directing agent (SDA) P123.

The produced nanorods were subsequently fluorescently labeled post-synthesis using the same pre-reaction solution as was added already in the synthesis step for S-MSP_1_. Thus, the negative surface charge of the NR-MSPs could also be preserved, and as a comparison to both S-MSPs, the NR-MSPs were PEI-functionalized both by surface grafting (NR-MSP-PEI graf.) and electrostatic adsorption (NR-MSP-PEI ads.). Furthermore, to investigate the surface charge effect of NR-MSPs, the NR-MSP-PEI graf. samples were further functionalized via either succinylation to yield negatively charged succinic acid groups [21,23] (NR-MSP-PEI-SUCC) or capping of the primary amines with uncharged acetyl groups [24] (NR-MSP-PEI-ACA). For both functionalization regimes, the PEI-functionalized particles were dispersed in DMF, into which either succinic or acetic anhydride was added in excess. The reaction suspension was agitated overnight. Next day, the particles were separated by centrifugation, washed with absolute ethanol and vacuum dried or directly dispersed into dimethyl sulfoxide (DMSO) at a concentration of 5 mg/ml for cellular experiments.

### Particle characterization methods

Thermogravimetrical analysis (Netzsch TG 209) was used to determine the amount of PEI added at temperature intervals of 170°C to 770°C. Successful modification of PEI and further derivatization with succinic acid or acetyl groups were further confirmed by zeta potential measurements (Malvern ZetaSizer NanoZS, Malvern Instruments Ltd., Worcestershire, UK). Full redispersibility of dried, extracted and surface-functionalized particles was confirmed by redispersion of dry particles in HEPES buffer at pH 7.2 and subsequent dynamic light scattering (DLS) measurements (Malvern ZetaSizer NanoZS). Scanning electron microscopy (SEM; Jeol JSM-6335 F (JEOL Ltd., Tokyo, Japan) for S-MSPs and Leo 1550 Gemini SEM (Zeiss, Oberkochen, Germany) for NR-MSPs) further confirmed the size, monodispersity, morphology and non-agglomerated state of the particles. The mesoscopic ordering of the nanoparticles was further confirmed by transmission electron microscopy (FEI Tecnai 12 TEM (FEI Co., Hillsboro, OR, USA) operating at 120 kV with a LaB6 filament and a 2 k × 2 k CCD camera) as well as powder XRD using a Kratky compact small-angle system (MBraun, Nottinghampshire, UK). The structural parameters related to the mesoporosity (surface area, pore size and pore volume) were determined by nitrogen sorption measurements (ASAP 2020 (Micromeritics Instrument Corp., Norcross, GA, USA) for NR-MSPs and Autosorb 1 (Quantachrome, Boynton Beach, FL, USA) for S-MSPs). Successful incorporation of fluorescein was determined by fluorescence spectrometry (Perkin Elmer LS 50B, PerkinElmer, Waltham, MA, USA) of particles dispersed in HEPES at a concentration of 0.5 mg/ml by excitation at 490 nm and determining the fluorescence intensity at wavelength 520 nm.

### Cell culturing, fluorescence-assisted cell sorting and cytotoxicity

#### Cell lines

HeLa cells and Caco-2 cells obtained from ATCC (Manassas, VA, USA) were maintained in DMEM medium (Sigma, St. Louis, MO, USA) supplemented with 10% fetal calf serum (BioClear, Wiltshire, UK), 2 mM L-glutamin, 100 U/ml penicillin and 100 μg/ml streptomycin at 37°C in a 5% CO_2_/95% O_2_ and 90% RH humidified atmosphere and handled under sterile conditions.

#### Cellular uptake by fluorescence-assisted cell sorting and confocal fluorescence microscopy

MSPs were suspended in a cell medium at different concentrations (1, 2 and 10 μg/ml) according to details given in Additional file 1. The amount of endocytosed particles inside the cells was analyzed by BD FacsCalibur flow cytometer (FL-I, BD Pharmingen, San Jose, CA, USA). The mean fluorescence intensity (MFI) of the cells at FL-1 channel was measured. The data were analyzed with BD CellQuest Pro™ software for the total amount of MSP uptake by 10,000 cells. GraphPad Prism 5.0 software was used for the statistical analysis of the results. The bar graphs in the figures represent mean values (±SD) from four or more independent experiments.

For microscopical studies, HeLa cells were seeded on a glass-bottom chamber slide (Lab-Tek™, Brendale, Australia) and incubated as explained in Additional file 1. The cells were viewed with Leica TCS SP5 confocal microscope (Leica Micosystems, Wetzlar, Germany; ×63 oil objective, 488 nm/514 nm/543 nm excitation).

#### Cell viability by WST-1 assay

HeLa cells were transferred to 96-well plates (9,000 cells/well) and allowed to attach and grow. After 24 h, the medium was removed and replaced with 100 μl medium containing different concentrations of MSPs (10 and 25 μg/ml). After incubation for 12 h, 10 μl of WST-1 (Roche Applied Science, Upper Bavaria, Germany) reagent was added to the cells, and further incubated for 90 min; after which, the 96-well plate was analyzed at a 430-nm wavelength in a Varioskan plate reader (Thermo Scientific, Logan UT, USA) to determine cell viability. Here, negative control (NC) is the cell media only without particles, NC DMSO-cell is the cell media with particle vehicle (DMSO), and positive control (PC) is with calyculin A (50 ng/ml) added to the cell media.

## Results

The characteristics of the produced particles in suspension form (0.5 mg/ml HEPES buffer) have been summarized in Table 1. The PEI amount (in weight percent with respect to the whole particle system) as determined from thermogravimetrical analysis was around 10 wt.% for all PEI-modified particles.

**Table 1:** Characteristics of silica nanoparticle suspensions (0.5 mg/ml) in HEPES buffer (25 mM, pH 7.2)

**Sample**	**Hydrodynamic Size (nm)**	**Zeta potential (mv)**	**Fluorescence intensity at *λ* ** **= 520 nm**
S-MSP_1_	687	−18	567
S-MSP_2_	875	≈0	392
NR-MSP	470	−16	626
S-MSP_1_-PEI ads.	692	22	216
S-MSP_2_-PEI graf.	569	33	203
NR-MSP-PEI ads.	298	15	266
NR-MSP-PEI graf.	388	23	227
NR-MSP-PEI-ACA	452	−12	170
NR-MSP-PEI-SUCC	389	−29	511

NR-MSP, rod-like mesoporous silica particles; NR-MSP-PEI-ACA, acetic anhydride-modified NR-MSP-PEI graf.; NR-MSP-PEI ads., 25 k poly(ethylene imine)-adsorbed NR-MSP; NR-MSP-PEI graf., poly(ethylene imine) surface-grown NR-MSP; NR-MSP-PEI-SUCC, succinic anhydride-modified NR-MSP-PEI graf.; S-MSP_1_, negatively charged spherical mesoporos silica nanoparticles; S-MSP_2_, neutrally charged spherical mesoporos silica nanoparticles; S-MSP_1_-PEI ads., 25 k poly(ethylene imine)-adsorbed S-MSP_1_; S-MSP_2_-PEI graf., poly(ethylene imine) surface-grown S-MSP_2_.

**Figure 3:**
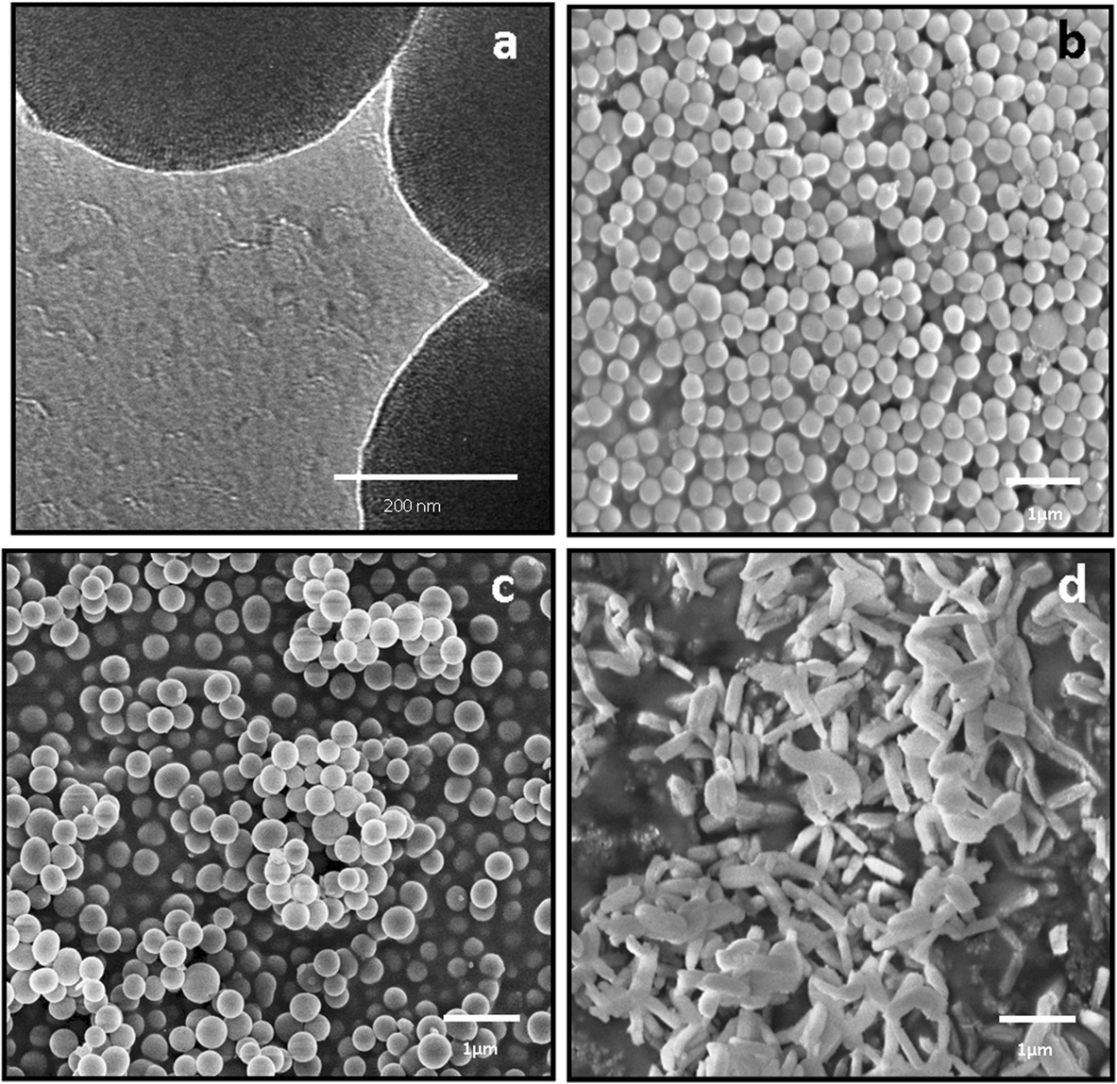
**Electron microscopy images of the produced mesoporous silica particles. (a)** TEM image of sample S-MSP_1_ illustrating the porous structure, **(b)** SEM image of samples S-MSP_1_, **(c)** S-MSP_2_ and **(d)** NR-MSP.

As a basis for comparison, these particular S-MSPs and NR-MSPs were chosen due to their similarity in size as determined from DLS methods on the nonfluorescent particles (see Figure S1 in Additional file 2), i.e., around 500 to approximately 600 nm by DLS as well as electron microscopy images (Figure 3). DLS for fluorescently labeled particles (Table 1) is carried out to confirm redispersibility of the particles in aqueous solvent at a physiologically relevant pH (7.2) after all processing steps (synthesis, drying, solvent extraction/calcination and surface functionalization), as judged by the quality of the measurement as well as the polydispersity index. The DLS (*z*-average) values listed in Table 1 are thus based on an average of three good quality measurements, thus confirming full redispersibility of all particles in HEPES buffer solution at pH 7.2, which was chosen because it is also used to buffer cell media in the cellular experiments. The DLS values listed in Table 1 cannot, however, be used to determine the hydrodynamic size in solution in this case as the fluorescent labeling may distort the calculations applied to derive size from light scattering measurements. Moreover, DLS cannot be correctly applied to derive the hydrodynamic size for non-spherical particles. Thus, the particle sizes and morphologies were rather determined by scanning electron microscopy (Figure 3). For the rod-like particles, the aspect ratio (AR) was determined to be 3 to 4 based on SEM analysis. Since no shape variety was observed for spherical particles, the aspect ratio is regarded as 1.

**Figure 4:**
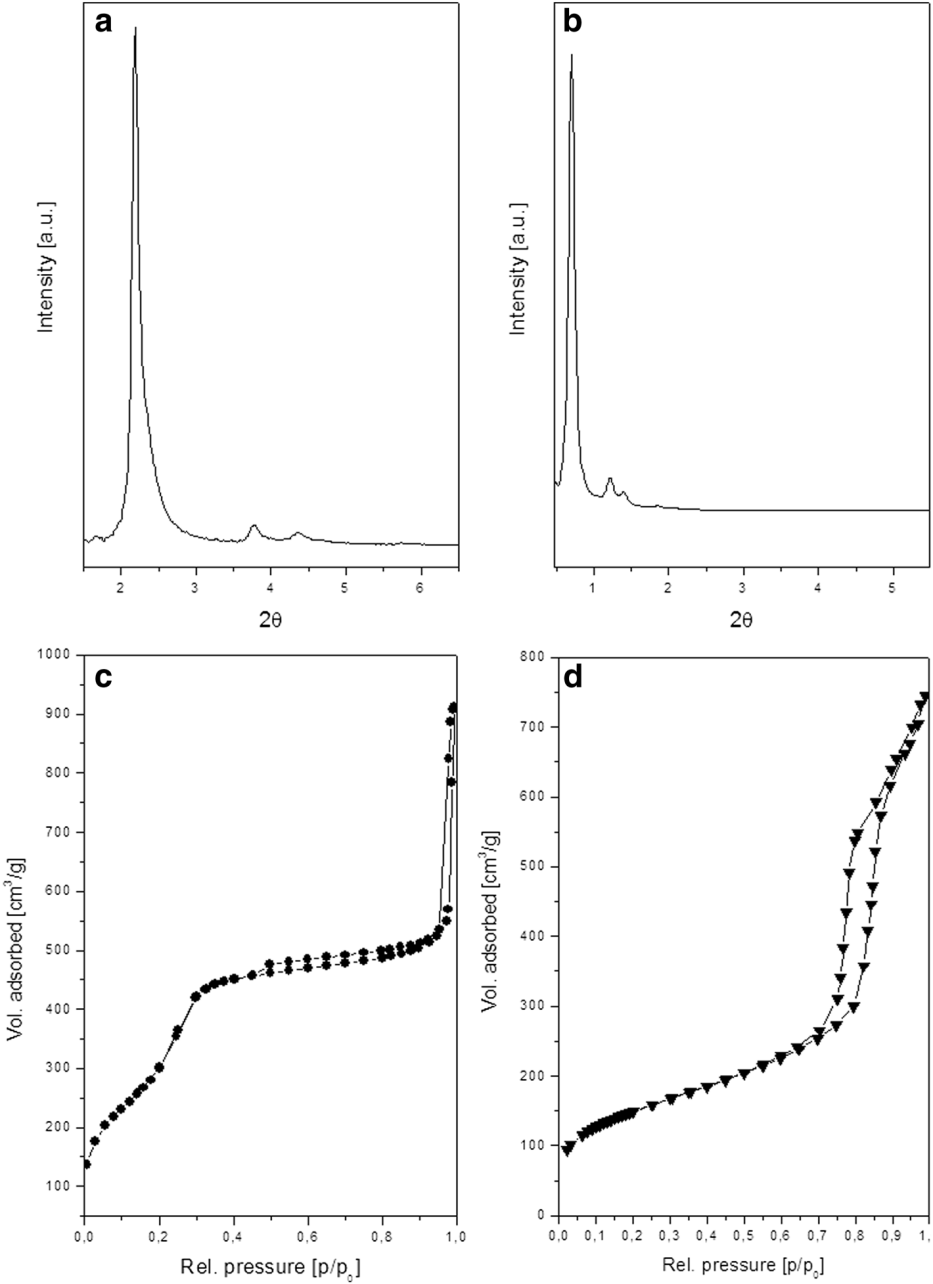
**Structural characteristics of the two different mesophases studied. (a)** Powder X-ray diffraction (PXRD) pattern of a typical S-MSP structure with hexagonally arranged pores (lattice spacing = 4.7 nm), **(b)** PXRD pattern of NR-MSP exhibiting hexagonal order with lattice spacing of 14.8 nm, **(c)** N_2_-sorption isotherm for S-MSP and **(d)** N_2_-sorption isotherm for NR-MSP, both of characteristic IUPAC type IV for mesoporous materials.

From the transmission electron microscopy image (Figure 3a), the mesoporous structure of the particle can also be deduced. The porous structure was also confirmed by powder X-ray scattering and nitrogen sorption measurements (Figure 4), from which the different pore size characteristics are also clearly observed. The S-MSPs were templated using cetyltrimethyl ammonium surfactants, typical of a MCM-41 synthesis, which in our reported case resulted in pore size of 3.5 nm (by Density functional theory (DFT)) and unit cell parameter of 4.7 nm (for S-MSP_1_ and S-MSP_2_). On the other hand, the SDA used for the synthesis of NR-MSP, the nonionic surfactant block-co-polymer P123 typical for the synthesis of SBA-15 materials, results in pore sizes of 7 nm and over in case additives are used [23,25]. In this case, the pore size of the NR-MSP is 11.3 nm (by DFT) with a unit cell parameter of 14.8 nm. Mesoporous silica nanorods have also been synthesized using CTAB as structure-directing agent with another nonionic surfactant, peregal Os-25, as co-template [26]. On an interesting note, rod-shaped SiC has also been prepared using uncalcined mesoporous silica SBA-15 as template [27]. The advantage with our applied synthesis regime, besides the exceptionally large pores that can be obtained, is the very short synthesis time (1 h) [23].

The fluorescent labeling of the particles regardless of method (co-condensation or post-synthesis grafting) were successful, as determined by fluorescence intensity measurements in HEPES buffer at the same concentrations (0.5 mg/ml; Table 1). On a related note, we want to highlight the alteration of fluorescence intensity upon surface functionalization when fluorescein is used as the fluorescent tag. Fluorescein, mostly used in the ‘activated’ form FITC, is a pH probe, and thus, both its absorption and fluorescence properties vary with pH as well as solvent [28]. This not only has implications for its intracellular localization upon quantification of cellular uptake as the pH in different cellular compartments differ, and for instance, in endo/lysosomes where particles would reside as a result of endocytotic uptake, the environment is acidic (pH 5 to 6). As is clearly illustrated in Table 1, surface functionalization, especially with acidic/basic groups such as amines or carboxylic acids (PEI and SUCC) which are the mostly used derivatizations when further bioconjugation is aimed for, also results in local pH changes on the nanoparticle surface. This has direct implications for differences in fluorescence intensity of the nanoparticles, despite being based on the same particle and measured at the same pH (here, in HEPES buffer at pH 7.2). This complicates fluorescence intensity-based quantification of cellular uptake as intracellular localization of the particles is not known at the time of detection whereby fluorescence intensity could also be measured at the right pH, and moreover, the localization for all particles within the same cell is not necessarily coinciding. Nevertheless, despite this drawback, fluorescein is the most widely used fluorescent tag due to its low cost, and these limitations in the absolute quantification based solely on fluorescence intensity are rarely taken into account. Consequently, we have chosen to mainly focus on percentage of positive cells in our cellular uptake studies as this value is not dependent on the absolute intensity of the particles.

**Figure 5:**
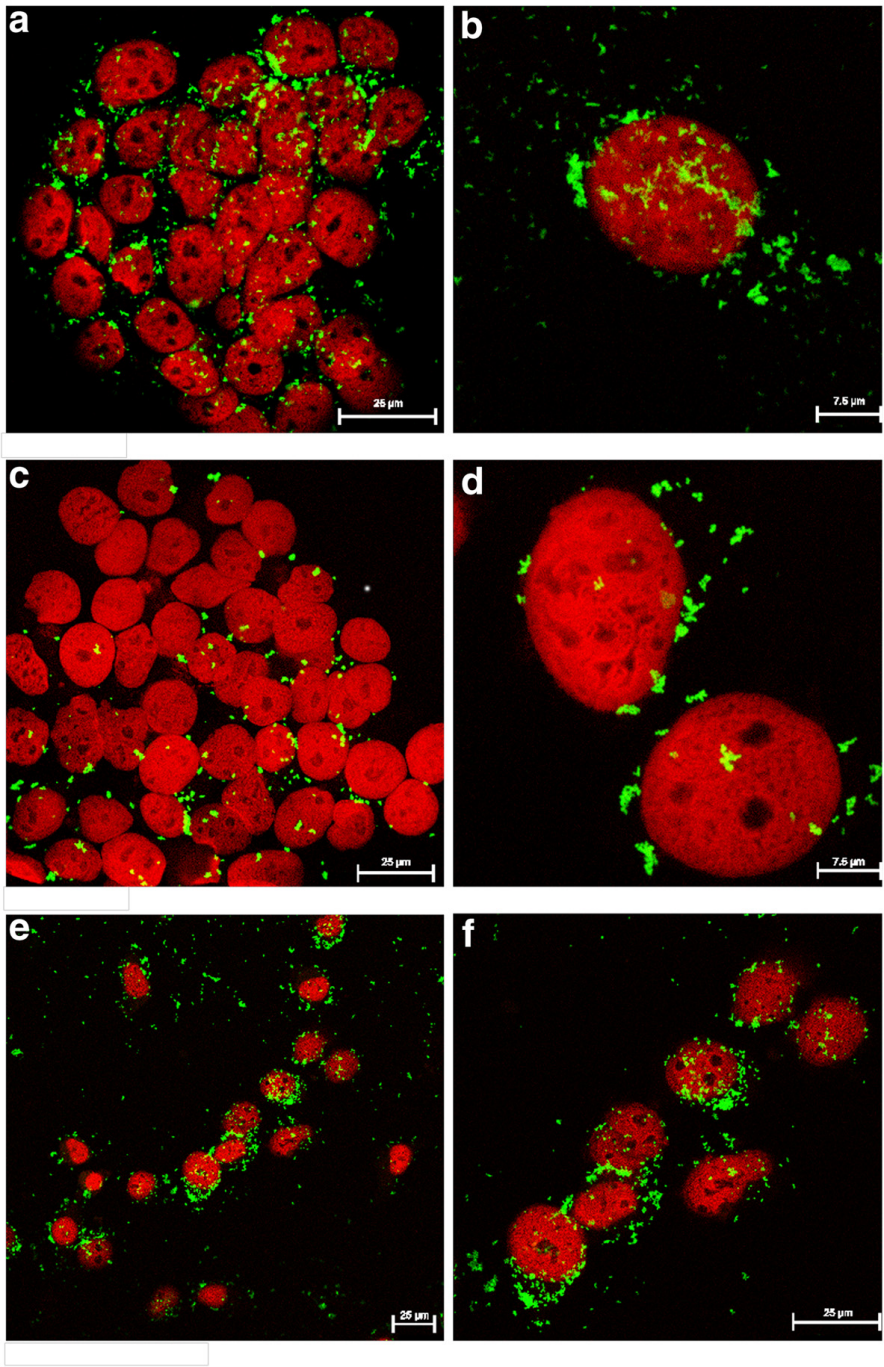
**Cellular uptake of particles as studied by confocal fluorescence microscopy.** Cellular uptake of uncoated rods **(a** and **b)** and spheres **(c** and **d)** vs PEI-functionalized S-MSPs **(e** and **f)** (spheres) in HeLa cells after 4 h incubation, resulting in different intracellular distribution patterns. All images are confocal sections thorough the nucleus.

Moreover, surface functionalization may also alter the intracellular localization of particles (Figure 5) as, for instance, PEI is commonly used as a nonviral gene transfection agent due to its ability to destabilize endosomal membranes and promote endosomal escape [29]. When compared to the uncoated spherical S-MSP_1_, indeed a more diffuse ‘spread-out’ pattern can be discerned for the PEI-coated particle, whereas a clearer intracellular aggregation of particles is seen when no PEI is present. This may be due to the fact that uncoated particles (Figure 5b) are more compartmentalized inside the cells as compared to their PEI-functionalized counterparts, which are more efficiently capable of escaping endo/lysosomes and consequently able to distribute inside the whole cell (cytoplasm). For the rod-like NR-MSPs, however, an almost fibrous pattern can be perceived, indicative of individual internalized rods (Figure 5).

### Cellular uptake of uncoated particles

**Figure 6:**
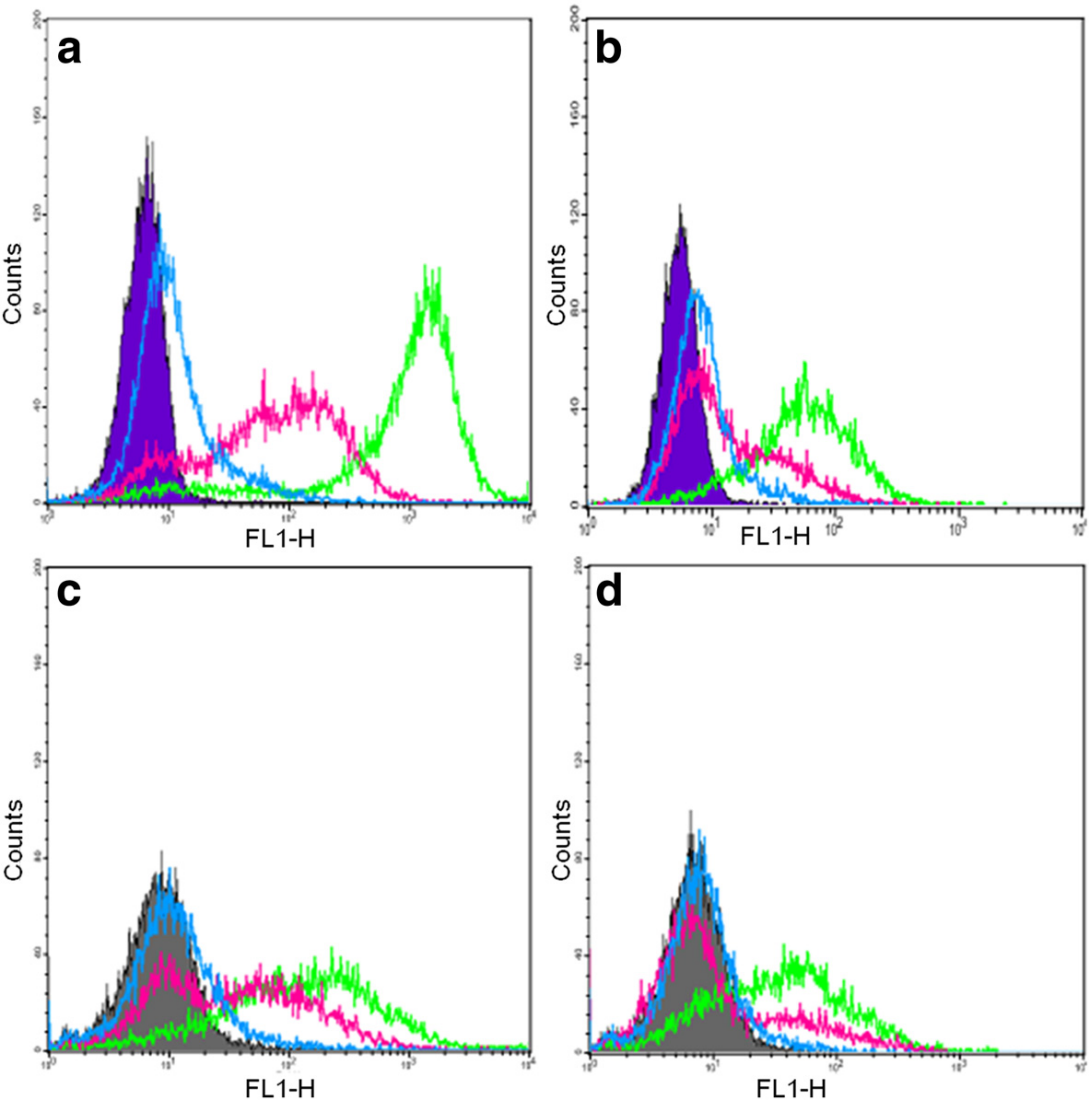
**Cellular uptake of uncoated MSPs in two different cancer cell lines after 4-h incubation. (a)** HeLa 10 μg/ml, **(b)** HeLa 2 μg/ml, **(c)** Caco-2 10 μg/ml and **(d)** Caco-2 2 μg/ml; NR-MSP (green), S-MSP_1_ (pink) and S-MSP-2 (blue). The shaded area is the control (no particles).

Cellular uptake was first studied on uncoated particles with different morphology in order to find out if shape effects could only result in different extents of internalization. The method of investigation was fluorescence-assisted cell sorting (FACS). The MFI values can be found in Additional files 3,4 and 5 for reference. We note that MFI could benefit from being normalized against particle-specific fluorescence before drawing any conclusions, and these values can also be found in Additional files 3 and 4. To broaden our observations, percentage of positive cells were furthermore determined for two different cell lines to investigate whether distinctions can be made based on cell origin also. Thus, two cancer cell lines were chosen for this purpose: HeLa cervical cancer cells and Caco-2 human epithelial colorectal adenocarcinoma cells. The Caco-2 cell lines are heterogenous and easily polarized when cultured under specific conditions, whereas HeLa are homogenous cells with a fibroblastic morphology. Cellular morphology could also influence uptake behavior, which is why we chose to study these two particular cell lines. Additionally, three different concentrations, 10, 2 and 1 μg/mL, were studied to find concentration-related differences. It is worth to mention that MSNs are frequently studied *in vitro* at concentrations up to 100 μg/mL, so our chosen concentrations are fairly low. As our particle sizes are also larger than in typical MSN studies (100 to approximately 200 nm), this also means that the number of particles used in our experiments is much lower than those in most studies to date. In order to detect internalized particles only, the extracellular fluorescence was quenched with trypan blue [30] in both microscopy and FACS studies. The resulting uptake of uncoated particles of different morphology in the two different cell lines at two different concentrations is illustrated in Figure 6.

**Figure 7:**
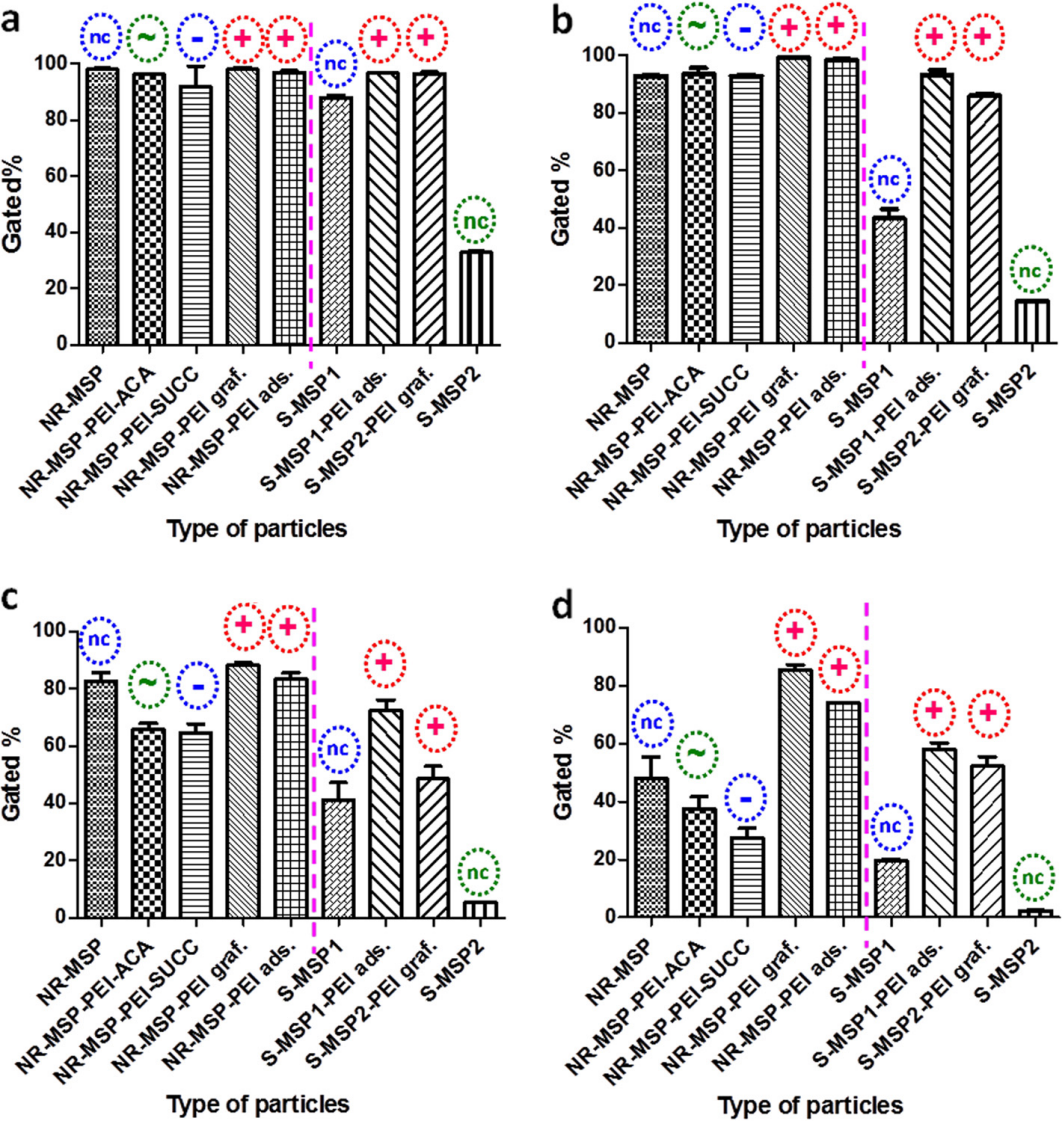
**Cellular uptake efficiency.** Cellular uptake of coated vs uncoated (nc) rods vs spheres in HeLa (**(a)** 10 μg/ml and **(b)** 2 μg/ml) vs Caco-2 (**(c)** 10 μg/ml and **(d)** 2 μg/ml) cell lines after 4 h incubation, as measured by FACS. Error bars represent SD (*n* ≥ 4). The pink dotted line distinguishes the rods from the spheres.

Clearly, there is a preference by both cell lines for rod-shaped particles (green histograms) as compared to spherical particles (Figure 6). This is less evident for HeLa cells at 10 μg/ml if only positive cell percentage values are regarded (see Figure 7) but more clearly so for the histogram plots (Figure 6). The observed high uptake is also corroborated by the microscopy results illustrated in Figure 5. Importantly, the differences in peak shift should also not exclusively be due to differences in fluorescence intensity between the particles as the inherent fluorescence for NR-MSP and S-MSP_1_ are similar (approximately 600; Table 1). It is interesting to note the bimodal peak distribution of S-MSP_1_, whereas the peak for S-MSP_2_ in all cases is not largely shifted from the control, indicating very low uptake. This distinction between the two spherical S-MSPs probably lies in the surface charge as S-MSP_1_ is negatively charged to resemble that of pristine silica materials, whereas S-MSP_2_ is a co-condensed material consisting of basic aminopropyl groups giving rise to positive charge at neutral pH. These abundant amino groups counteract the acidic silanol groups responsible for the negative charge of silica surfaces, thus resulting in a net neutral charge under the studied conditions. As the NR-MSPs were post-synthesis-functionalized with FITC-APTES in just enough amount to yield a sufficient fluorescent labeling, the surface of this material also resembles that of pure silica (and S-MSP_1_). It is noteworthy that previous MSN reports studying rod-like particles in a biological setting have also been fluorescently labeled according to similar protocols, so our S-MSP_1_ and NR-MSP results can advantageously be compared to those. Indeed, the same trend that rod-like particles is preferred over spherical ones is observable in our results, perfectly in line with earlier reports as discussed in the ‘Background’ section. The difference in uptake between the spherical particles (S-MSP_1_ and S-MSP_2_), observed in both cell lines, is also interesting. It has been proposed that serum protein adsorption onto particles, coronation, leads to enhanced unspecific cellular uptake and that high charge, whether negative or positive, results in increased protein adsorption. In light of this, the two negatively charged particles S-MSP_1_ and NR-MSP could be subject to higher protein adsorption (from the cell media), which could lead to increased cellular uptake. On the other hand, a near-neutral charge such as the one observed for S-MSP_2_ also indicates diminished electrostatic dispersion stabilization, which could lead to particle aggregates that are too large for the cells to take up. Furthermore, as the particles are uncoated, no steric stabilization is provided, and thus, this particular MSP system may be susceptible to aggregation in the physiological environment.

### Cellular uptake of organically modified particles

Organic modification is almost exclusively required to obtain desirable properties for a MSN system in order to provide for eligible biobehavior. The added value in organic modification may include dispersion stability, surface charge tuning, stealth properties, anchoring points for biofunctionalization (antibodies, proteins, peptides, targeting ligands and drug molecules), increased hydrophilicity, molecular gate-keeping, stimuli responsiveness, controlled cleavage of surface-bound coating or moieties, and so forth. On a less sophisticated level, it is generally believed that a net positive charge is beneficial in maximizing cellular uptake due to attraction to the negatively charged cell membrane. Thus, functionalization with positively charged surface groups is frequently applied to enhance cellular uptake. This is also the primary reason why we decided to functionalize our particles with the cationic polyelectrolyte PEI. Aside from enhanced cellular uptake, the branched structure of PEI also offers a higher amount of terminal primary amino groups than possible to attain via conventional amino functionalization, simultaneously providing increased electrostatic suspension stability to the system and possibly introducing pH-dependent molecular gate properties along with enabling endosomal escape ability, both of which would be utilized when carrying drug cargo for intracellular release [31]. To investigate the effect on cellular uptake upon derivatization of the PEI layer, we also capped the terminal primary amino groups with either uncharged acetyl groups or acidic (negatively charged under neutral conditions) succinic acid groups. The uptake in terms of positive cell percentage of the whole series of particles with (or without) different functionalization is presented in Figure 7.

**Figure 8:**
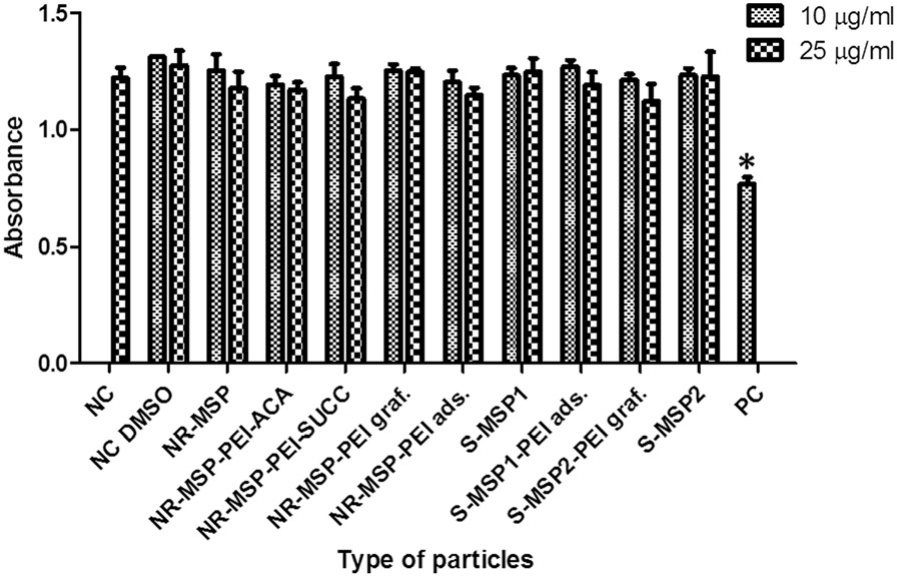
**Cell viability.** Viability of HeLa cells incubated with rod-like and spherical mesoporous silica nanoparticles at 12 h, assessed by a WST-1 assay. Error bars represent SD (*n* ≥ 3). Statistical analysis was made by ANOVA followed by a Dunnett's multiple comparison post-test. All data sets were compared with a negative cytotoxicity control of cell media with a particle vehicle (DMSO). The level of significance was set at a probability of *p < 0.001. NC, negative control; PC, positive control (calyculin A).

For HeLa cells, the uptake is efficient to a degree that no differences between surface-modified NR-MSPs could be detected even at concentrations as low as 2 μg/ml, whereas a decreased uptake of (uncoated) spheres was observed also at 10 μg/ml. For all other particles, the uptake was virtually 100% for both concentrations. For Caco-2 cells, the differences were clearer and followed what could be expected regarding surface-charge-induced differences. Capping the charge with uncharged groups reduces the uptake, but a negative charge seems to reduce it even more. This effect is even more evident from the graph of normalized fluorescence intensity vs type of particles in Figure S3 in Additional file 4, where MFI values from FACS have been normalized against particle-specific fluorescence in suspension. This observed effect is probably due to the fact that the absolute charge is not neutral, but still, a competition between negatively charged silanols on the underlying silica surface and secondary and tertiary amine groups in the PEI layer results in a net (overall) neutral effective charge. As this net effective charge recorded at neutral pH was even slightly negative, the capping of the terminal primary amines seems to have been very effective as it has been able to shield the positive charge. As the terminal acetyl groups are not charged, this ‘charge capping’ possibly serves to reduce the enhancement in uptake observed for positively charged particles. This kind of charge capping consequently has also been used to reduce the toxicity associated with high molecular weight PEI when used as a gene carrier [24]. To account for this well-known PEI toxicity, as well as the notation that surface modification could have a profound impact on the cytotoxicity of the particles, cellular viability was also determined for the whole particle series (Figure 8).

**Figure 9:**
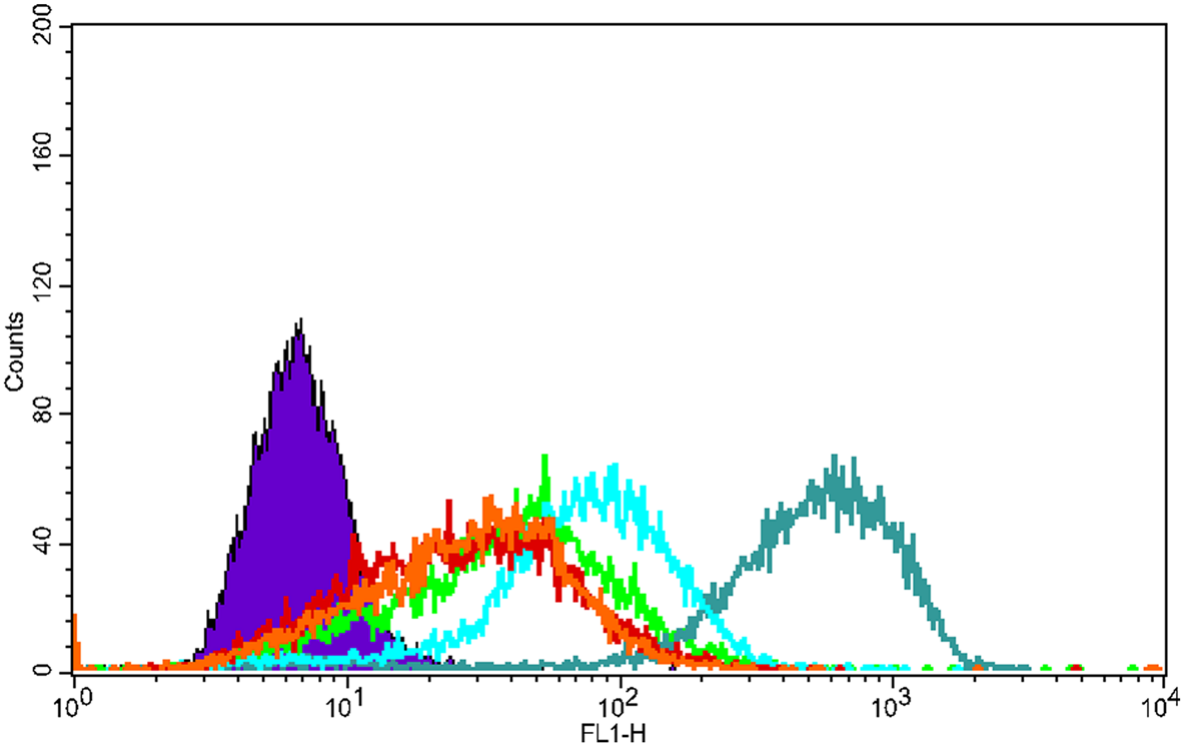
**Charge-induced differences in uptake.** Incubation of NR-MSPs in HeLa cells at a low concentration (1 μg/ml), NR-MSP (green, 83.6%), NR-MSP-PEI-ACA (red, 62.4%), NR-MSP-PEI-SUCC (orange, 69.6%), NR-MSP-PEI adsorbed (light blue, 93.2%), NR-MSP-PEI grafted (teal, 97.9%) and control (violet).

On the other hand, the succinylation provides for terminal negatively charged groups on the organic layer which, together with the secondary and tertiary amine groups in the PEI layer (or any residual primary amines), make up an organic layer consisting of both negative and positive charges. Not only has succinylation of branched PEI shown to lead to a ten-fold decrease in toxicity observed for pure PEI [24] but such zwitterionic functionalization has also been suggested to minimize serum protein adsorption [32], which could otherwise lead to increased unspecific uptake. Thus, it may not be the negative charge as such that reduces the uptake but the zwitterionic nature of the organic layer [33]. This is also supported by the higher uptake observed for the negatively charged uncoated particles (Figures 6 and 7c,d) as compared to the net neutral S-MSP_2_ consisting of a surface with negatively charged silanols and positively charged aminopropyl groups, which hardly exhibits any uptake at all. In Figure 7, no distinctions for the rod-like particles can be deduced as they are all taken up almost in 100% of the cells, but for HeLa cells at even lower concentrations (1 μg/ml), the charge-induced differences also start to occur as shown in the histogram presented in Figure 9 with the same trend, i.e., the positively charged rod-like particles are taken up to a greater extent. One interesting notation from both Figure 9 and the MFI values in Additional files 3,4 and 5 (normalized or not) is that the surface-polymerized PEI seems to be much more efficient in inducing cellular uptake of the rod-shaped MSNs as compared to the more commonly applied electrostatic adsorption of commercially available PEI. Thus, not only purely charge-induced effects but also the functionalization regime seems to be useful in fine-tuning the cellular uptake at lower doses as a ‘secondary’ uptake regulator, if desired.

## Discussion

Our observed enhanced uptake of the rod-like MSPs is in line with previous reports on rod-like MSN biobehavior. These previous reports have mainly concentrated on non-functionalized MSNs aside from the fluorescent labeling (i.e., comparable to our S-MSP_1_ and NR-MSP) to render the particles observable by confocal (fluorescence) microscopy and other applied fluorescence-based characterization techniques. Meng et al. [13] investigated the aspect ratio effect on cellular uptake rate and amount, and found that particles with an AR of 2.1 to 2.5 were taken up in greater quantities than shorter (1.5 to 1,7) and longer (4 to 4.5) rods as well as spheres (AR = 1), which seem consistent with earlier reports on organic nanoparticles for which AR ~ 3 was found to be the most efficient in terms of cellular internalization [4]. The aspect ratio of our studied NR-MSP is around 3 to 4. Meng et al. stated that their observations implied that a cellular mechanism capable of discerning and responding to rod length was operative, and compared this observation to that of Champion and Mirtagotri [34], who studied the phagocytosis mechanism of non-spherical polymer particles in macrophages and concluded that it was the local geometry (angle of curvature) at the point of contact with the cell membrane that initiated phagocytosis, not the overall AR. It is worth to mention that the two mechanisms of uptake studied in the two separate studies were different, though Meng et al. studied macropinocytosis and the effect of particle shape on this particular cellular uptake route in two cancer cell lines (HeLa and A549) [13]. Huang et al. also studied the effect of MSNs of different ARs (1, 2 and 4) on uptake amounts and rates in a human melanoma (A357) cell line as well as AR impacts on cellular function including cell proliferation, apoptosis, cytoskeleton formation, adhesion and migration [12]. They found that increasing AR gave rise to increased uptake whereas long rod-shaped particles could cause reorganization of the cytoskeleton, and ascribed this observation to the more efficient endocytosis of rod MSNs than spheres [12]. Whereas Meng et al. found the behavior to be similar between the two cancer cell lines studied [13], Trewyn et al. found slight differences between cell lines when comparing a cancer cell line (Chinese hamster ovarian) to a human fibroblast cell line [16]. In our current investigation between two cancer cell lines, the overall trends seem to be similar but with different dose-dependencies. Huang et al. [14] studied the effect of uncoated and PEGylated MSNs with two different ARs (1.5 and 5) on biodistribution, clearance and biocompatibility *in vivo*, whereby they found that the AR affected the clearance rate in that short rod MSNs (AR ~ 1.5) had a more rapid clearance rate than the long rod MSNs (AR ~ 5). No profound *in vivo* toxicity (based on hematology, serum biochemistry and histopathology) was found, in line with their previous report where MSN concentrations as high as 1,000 mg/kg were used [35]. Another useful means of predicting *in vivo* compatibility when aiming for circulating nanoparticles is determining their hemolytic activity, whereby Yu et al. have investigated pure and amino-functionalized porous and non-porous silica nanoparticles of ARs 2, 4 and 8 and their cytotoxicity effects on macrophages (RAW 264.6), lung carcinoma cells (A549) and hemolysis of human erythrocytes [15]. The pure silica particles showed a porosity- and shape-dependency on hemolytic activity, with the porous MSNs with higher AR exhibiting reduced hemolytic activity, whereas for the amino-functionalized particles, the zeta potential (effective surface charge) was decisive [15]. On this note, we want to stress that as we have particles of distinctively different pore sizes (3 to 4 nm vs 11 to 12 nm), this might give rise to differences in hemolytic activity as it has also been pointed out earlier that porosity is a decisive property due to lower exposure of silanol groups to the cell membrane of a porous surface [36]. However, as surface modification eliminates this source of interaction, the porosity effect might be of subordinate importance for surface-modified MSPs. In the study by Yu et al., surface charge and porosity also governed cellular toxicity, whereas AR did not have any reported effect [15]. Also in our current study, no differences in cytotoxicity were observed at doses relevant for our experiments and higher (2.5 times our maximum used dose). We also note that the two different pore sizes of our chosen MSPs are rationalized based on applicability for different types of therapeutic cargo, whereby the 3- to 4-nm pores are ideal for the loading of small-molecular drugs, whereas the large-pore materials would also be suitable for carrying of biomacromolecules such as proteins (antibodies, enzymes, polypeptides and so on) and genes [37,38]. Finally, based on the troublesome nature of the fluorescein label, a more quantitative/absolute approach to studying the shape effect on nanoparticle uptake with nanomedical prospects and the efficiency of different particles in cargo delivery could constitute a more correlative approach. To date, this has been successfully measured in the extent of causing cytotoxicity upon delivery of the chemotherapeutic agent camptothecin or paclitaxel [13] or the efficacy of GFP knockdown [39] upon siRNA delivery, and similar approaches will also be pursued in our ongoing and future studies.

## Conclusions

Porous silica particles of spherical and rod-like morphologies were studied for cellular uptake efficiency in two different cancerous cell lines for potential applications as nanomedical drug delivery carriers. According to the obtained results, both rod-like and spherical particles were readily internalized by HeLa cells with slight shape and charge-induced differences, whereas in Caco-2 cells, rod-shaped particles were internalized more efficiently. The difference was most pronounced for uncoated particles in both cell lines, whereby higher charge (S-MSP_1_) also induced higher uptake. A net positive charge (PEI) enhanced uptake regardless of shape and cell line. At lower doses, surface charge could be used to fine-tune the uptake even in HeLa cells, whereby higher charge (+/−) results in higher uptake over net neutral charge and positive over negative. At higher concentrations, the surface charge effect is overridden in HeLa cells, and rods are taken up despite coating or not, whereas for spheres and Caco-2 cells, distinctions can still be made. Uptake studies performed *in vitro* in different cell lines show that along with particle shape and surface functionalization, cellular origin and features may also influence the uptake of particles in cells. As shape seems to influence uptake in a cell-dependent manner, shape engineering could potentially be used as a tool for enhancing nanoparticle-mediated delivery.

## Competing interests

The authors declare no competing interests.

## Authors' contributions

DSK carried out the synthesis, functionalization and characterization of mesoporous silica nanoparticles, analyzed and interpreted characterization data and some part of the *in vitro* test results and has been involved in drafting and designing of the manuscript. DD carried out the *in vitro* tests such as cell viability, confocal microscopy and some part of the FACS studies, analyzed and interpreted the obtained *in vitro* results and has been involved in drafting and designing of the manuscript. RS studied the cellular uptake of sphere and rod-like MSPs at different concentrations in HeLa cell lines adopting FACS. EMJ carried out the synthesis and characterization of mesoporous silica nanorods and has been involved in drafting the manuscript. NR carried out the preliminary uptake and cytotoxicity studies of differently functionalized nanorods. MO designed and characterized the mesoporous nanorods in addition to revising the manuscript critically and contributing with important intellectual content. JEE provided biological lab facilities along with CS and DMT. CS has been involved in revising the manuscript critically for important intellectual content. DMT has been involved in providing the cell cultures and reagents for toxicity testing and revising the manuscript critically for important intellectual content. JMR conceived, designed and coordinated the study and drafted/wrote the manuscript. All authors read and approved the final manuscript.

## Supplementary Material

Additional file 1 Supplementary methods. Click here for file

Additional file 2 **Figure S1** DLS measurements of nonfluorescent NR-MSPs (red) and S-MSPs (green) measured in water after SDA removal and drying. Click here for file

Additional file 3 **Figure S2** MFI values from FACS for HeLa cells incubated with 10 and 2 μg/mL MSPs for 4 h. The lower graphs have been normalized against pure particle suspension fluorescence values measured at 530 nm in HEPES buffer at pH 7.2. Click here for file

Additional file 4 **Figure S3** MFI values from FACS for Caco-2 cells incubated with 10 and 2 μg/mL MSPs for 4 h. The lower graphs have been normalized against pure particle suspension fluorescence values measured at 530 nm in HEPES buffer at pH 7.2. Click here for file

Additional file 5 **Figure S4** MFI values from FACS for HeLa cells incubated with 1 μg/mL MSPs for 4 h. The right graph has been normalized against pure particle suspension fluorescence values measured at 530 nm in HEPES buffer at pH 7.2. Click here for file

## References

[B1] Niemeyer CM Nanoparticles, proteins, and nucleic acids: biotechnology meets materials science Angew Chem Int 2001 40 4128 4158 10.1002/1521-3773(20011119)40:22<4128::AID-ANIE4128>3.0.CO;2-S 29712109

[B2] Mitagotri S In drug delivery, shape does matter Pharmaceutical Research 2009 26 1 232 234 10.1007/s11095-008-9740-y 18923811

[B3] Venkataraman A Hedrick JL Ong ZY Yang C Rachel Ee PL Hammond PT Yang YY The effects of polymeric nanostructure shape on drug delivery Adv Drug Delivery Rev 2011 63 1228 1246 10.1016/j.addr.2011.06.016 21777633

[B4] Gratton SEA Ropp PA Pohlhaus PD Luft JC Madden VJ Napier ME DeSimone JM The effect of particle design on cellular internalization pathways PNAS 2008 105 33 11613 11618 10.1073/pnas.0801763105 18697944 PMC2575324

[B5] Mitragotri S Designer polymer particles for drug delivery [abstract] Tools for ADMET and Pharmaceutical Nanotechnology: September 18–20 2011; Helsinki. 2011 2011 Helsinki: University of Helsinki

[B6] Decuzzi P Pasqualini R Arap W Ferrari M Intravascular delivery of particulate systems: does geometry really matter? Pharmaceutical Research 2009 26 1 235 243 10.1007/s11095-008-9697-x 18712584

[B7] Rosenholm JM Sahlgren C Lindén M Towards intelligent, targeted drug delivery systems using mesoporous silica nanoparticles – opportunities & challenges Nanoscale 2010 2 1870 1883 10.1039/c0nr00156b 20730166

[B8] Popat A Hartono SB Stahr F Liu J Qiao SZ Lu GQ Mesoporous silica nanoparticles for bioadsorption, enzyme immobilization, and delivery carriers Nanoscale 2011 3 2801 2818 10.1039/c1nr10224a 21547299

[B9] Li Z Barnes JC Bosoy A Stoddart JF Zink JI Mesoporous silica nanopartilces in biomedical applications Chem Soc Rev 2012 10.1039/c1cs15246g 22216418

[B10] Lee JE Lee N Kim T Kim J Hyeon T Multifunctional mesoporous silica nanocomposite nanoparticles for theranostic applications Acc Chem Res 2011 44 10 893 902 10.1021/ar2000259 21848274

[B11] Rosenholm JM Mamaeva V Sahlgren C Lindén M Nanoparticles in targeted cancer therapy: mesoprous silica nanopartilces entering preclinical development stage Nanomedicine 2012 7 111 120 10.2217/nnm.11.166 22191780

[B12] Huang X Teng X Chen D Tang F He J The effect of the shape of mesoporous silica nanoparticles on cellular uptake and cell function Biomaterials 2010 31 438 448 10.1016/j.biomaterials.2009.09.060 19800115

[B13] Meng H Yang S Li Z Xia T Chen J Ji Z Zhang H Wang X Lin S Huang C Zhou ZH Zink JI Nel AE Aspect ratio determines the quantity of mesoporous silica nanoparticle uptake by a small GTPase-dependent macropinocytosis mechanism ACS Nano 2011 5 6 4434 4447 10.1021/nn103344k 21563770 PMC3125420

[B14] Huang X Li L Liu T Hao N Liu H Chen D Tang F The shape effect of mesoporous silica nanoparticles on biodistribution, clearance, and biocompatibilty in vivo ACS Nano 2011 5 7 5390 5399 10.1021/nn200365a 21634407

[B15] Yu T Malugin A Ghandehari H Impact of silica nanoparticle design on cellular toxicity and hemolytic activity ACS Nano 2011 5 5717 5728 10.1021/nn2013904 21630682 PMC3238493

[B16] Trewyn BG Nieweg JA Zhao Y Lin VSY Biocompatible mesoporous silica nanoparticles with different morphologies for animal cell membrane penetration Chem Eng J 2008 137 23 29 10.1016/j.cej.2007.09.045

[B17] He Q Shi J Chen F Zhu M Zhang L An anticancer drug delivery system based on surfactant-templated mesoporous silica nanoparticles Biomaterials 2010 31 12 3335 3346 10.1016/j.biomaterials.2010.01.015 20106517

[B18] Rosenholm JM Meinander A Peuhu E Niemi R Eriksson JE Sahlgren C Lindén M Targeting of porous hybrid silica nanoparticles to cancer cells ACS Nano 2009 3 1 197 206 10.1021/nn800781r 19206267

[B19] Rosenholm JM Penninkangas A Lindén M Amino-functionalization of large-pore mesoscopically ordered silica by a one-step hyperbranching polymerization of a surface-grown polyethyleneimine Chem Commun 2006 37 3909 3911 10.1039/b607886a 17268667

[B20] Rosenholm JM Lindén M Wet-chemical analysis of surface concentration of accessible groups on different amino-functionalized mesoporous SBA-15 silicas Chem Mater 2007 19 5023 5034 10.1021/cm071289n

[B21] Rosenholm JM Duchanoy A Lindén M Hyperbranching surface polymerization as a tool for preferential functionalization of the outer surface of mesoporous silica Chem Mater 2008 20 1126 1133 10.1021/cm7021328

[B22] Johansson EM Ballem MA Córdoba JM Odén M Rapid synthesis of SBA-15 rods with variable lengths, widths, and tunable large pores Langmuir 2011 27 4994 4999 10.1021/la104864d 21413751

[B23] Bergman L Rosenholm JM Öst AB Duchanoy A Kankaanpää P Heino J Lindén M On the complexity of electrostatic suspension stabilization of functionalized silica nanoparticles for biotargeting and –imaging applications J Nanomaterials 2008 10.1155/2008/712514

[B24] Zintchenko A Philipp A Dehshahri A Wagner E Simple modifications of branched PEI lead to highly efficient siRNA carriers with low toxicity Bioconjugate Chem 2008 19 1448 1455 10.1021/bc800065f 18553894

[B25] Johansson EM Córdoca JM Odén M Effect of heptane addition on pore size and particle morphology of mesoporous silica SBA-15 Microporous Mesoporous Mater 2010 133 66 74 10.1016/j.micromeso.2010.04.016

[B26] Li M Wang N Liang Y Zhang J Preparation of monodisperse short rod-like mesoporous silica Chinese J Mat Res 2006 20 2 181 185

[B27] Selvan ST Aldeyab SS Zaidi JSM Arivuoli D Ariga K Mori T Vinu A Preparation and characterization of highly ordered mesoporous SiC nanoparticles with rod shaped morphology and tunable pore diameters J Mater Chem 2011 21 8792 8799 10.1039/c1jm10545k

[B28] Cook A Le A The effect of solvent and pH on the fluorescence excitation and emission spectra of solutions containing fluorescein J Phys Chem Lab 2006 10 44 49

[B29] Boussif O Lezoualc'h F Zanta MA Mergny MD Scherman D Demeneix B Behr JP A versatile vector for gene and oligonucleotide transfer into cells in culture and in vivo: polyethylenimine Proc Natl Acad Sci U S A 1995 92 16 7297 7301 10.1073/pnas.92.16.7297 7638184 PMC41326

[B30] Sahlin S Hed J Rundquist I Differentation between attached and ingested immune complexes by a fluorescence quenching cytofluorometric assay J Immunoll Methods 1983 60 115 124 10.1016/0022-1759(83)90340-X 6406600

[B31] Rosenholm JM Peuhu E Eriksson JE Sahlgren C Lindén M Targeted intracellular delivery and release of hydrophobic agents using mesoporous hybrid silica nanoparticles as drug carrier systems Nano Letters 2009 9 3308 3311 10.1021/nl901589y 19736973

[B32] Choi HS Liu W Misra P Tanaka E Zimmer JP Kandapallil B Bawendi MG Frangioni JV Renal clearance of quantum dots Nat Biotechnol 2007 25 1165 1170 10.1038/nbt1340 17891134 PMC2702539

[B33] Verma A Stellacci F Effect of surface properties on nanoparticle-cell interactions Small 2010 6 12 21 10.1002/smll.200901158 19844908

[B34] Champion JA Mitragotri S Shape induced inhibition of phagocytosis of polymer particles Pharmaceutical Research 2009 26 1 244 249 10.1007/s11095-008-9626-z 18548338 PMC2810499

[B35] Liu TL Li LL Teng X Huang XL Liu HY Chen D Ren J He JQ Tang FQ Single and repeated dose toxicity of mesoporous hollow silica nanoparticles in intravenously exposed mice Biomaterials 2011 32 1657 1668 10.1016/j.biomaterials.2010.10.035 21093905

[B36] Lin YS Haynes CL Impacts of mesoporous silica nanoparticle size, pore ordering, and pore integrity on hemolytic activity JACS 2010 132 13 4834 4842 10.1021/ja910846q 20230032

[B37] Rosenholm JM Sahlgren C Lindén M Multifunctional mesoporous silica nanoparticles for combined therapeutic, diagnostic and targeted action in cancer treatment Current Drug Targets 2011 12 8 1166 1186 10.2174/138945011795906624 21443474

[B38] Rosenholm JM Zhang J Sun W Gu H Large-pore mesoporous silica-coated magnetite core-shell nanocomposites and their relevance for biomedical applications Microporous Mesoporous Materials 2011 145 1–3 14 20

[B39] Kolhar P Doshi N Mitragotri S Polymer nanoneedle-mediated intracellular drug delivery Small 2011 7 14 2094 2100 10.1002/smll.201100497 21695782

