# Supplementary material for: Shape engineering vs organic modification of inorganic nanoparticles as a tool for enhancing cellular internalization
Source: Nanoscale Res Lett. 2012 Jul 1;7(1):358. doi: 10.1186/1556-276X-7-358 (PMC3519764; doi:10.1186/1556-276X-7-358)
Supplement: Additional file 1 — Supplementary methods. [file 1556-276X-7-358-S1.docx]

**Supplementary Methods (Additional file 1)**

*Spherical mesoporous silica particles*: **S-MSP_1_** were synthesized according to the protocol described in reference [17] with slight modifications as follows. Absolute ethanol was added to aqueous basic reaction solution with 20 v/v-% of reaction solution as co-solvent to promote a spherical shape[18]. Cetyltrimethylammonium bromide (CTAB) and etraethylorthosilicate (TEOS) were mixed to the basic aqueous reaction solution as structure-directing agent (SDA) and silica source, respectively. The reaction was conducted in a round-bottom flask at 80 ^0^C with condenser for overnight. The molar composition in synthesis solution was 1 TEOS: 1.23 x 10-4 CTAB: 0.31 NaOH: 73.3 EtOH: 948.93 H2O. After overnight reaction of S-MSP1 the SDA was removed by sonication extraction three times in ethanolic NH_4_NO_3_ solution.

**S-MSP_2_** were prepared similarly to the S-MSP_1_ synthesis, with the exception that methanol was used as co-solvent and cetyltrimethylammoniumchloride (CTAC) as SDA. Thus, also the methoxy versions of the silanes were used, with tetramethyl orthosilicate (TMOS) and aminoproplylrimethoxysilane (APTMS) as silica sources, A typical synthesis solution consisted of a molar ratio of 0.9 TMOS: 0.1 APTMS: 1.27 CTAC: 0.26 NaOH: 1439 MeOH: 2560 H_2_O. After the overnight synthesis, the particles were separated by centrifugation, vacuum dried and subsequently SDA was removed by sonication extraction three times in acidic (HCl) ethanol.

*Cellular uptake by fluorescence-assisted cell sorting (FACS) and confocal fluorescence microscopy:* MSPs were suspended in cell medium at different concentrations (1 µg/ml, 2 µg/ml and 10 µg/ml).After 30 min sonication in water bath, the medium containing the particles or control medium was applied to the 50-70% confluent cells and incubated for 4 h at 37 °C. The cells were trypsinized and the extracellular fluorescence was quenched by resuspension in 200 µg/ml trypan blue (Fluka) for 7 min at room temperature. The cells were washed once and resuspended in PBS.

For microscopical studies HeLa cells were seeded on glass-bottom chamber slide (Lab-Tek™) and incubated with particles for 4 h. After incubation cell media was removed and cells were washed with PBS and treated with trypan blue (200 µg/ml). Cells were washed and fixed with 4% paraformaldehyde, nuclei were stained using propidium iodide (1 mg/ml, Invitrogen) and finally coverslips were mounted using ProLong® Gold antifade reagent. The cells were viewed with Leica TCS SP5 confocal microscope (63X oil objective, 488nm/ 514nm/ 543nm excitation).
