# Supplementary figures and images for: Shape engineering vs organic modification of inorganic nanoparticles as a tool for enhancing cellular internalization
Source: Nanoscale Res Lett. 2012 Jul 1;7(1):358. doi: 10.1186/1556-276X-7-358 (PMC3519764; doi:10.1186/1556-276X-7-358)

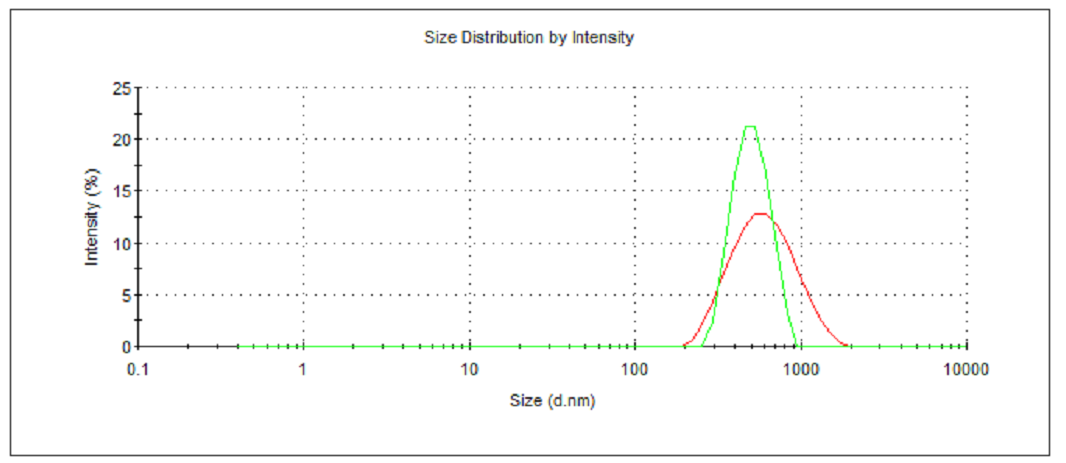

Supplement: Additional file 2 — Figure S1 DLS measurements of nonfluorescent NR-MSPs (red) and S-MSPs (green) measured in water after SDA removal and drying. [file 1556-276X-7-358-S2.tiff]

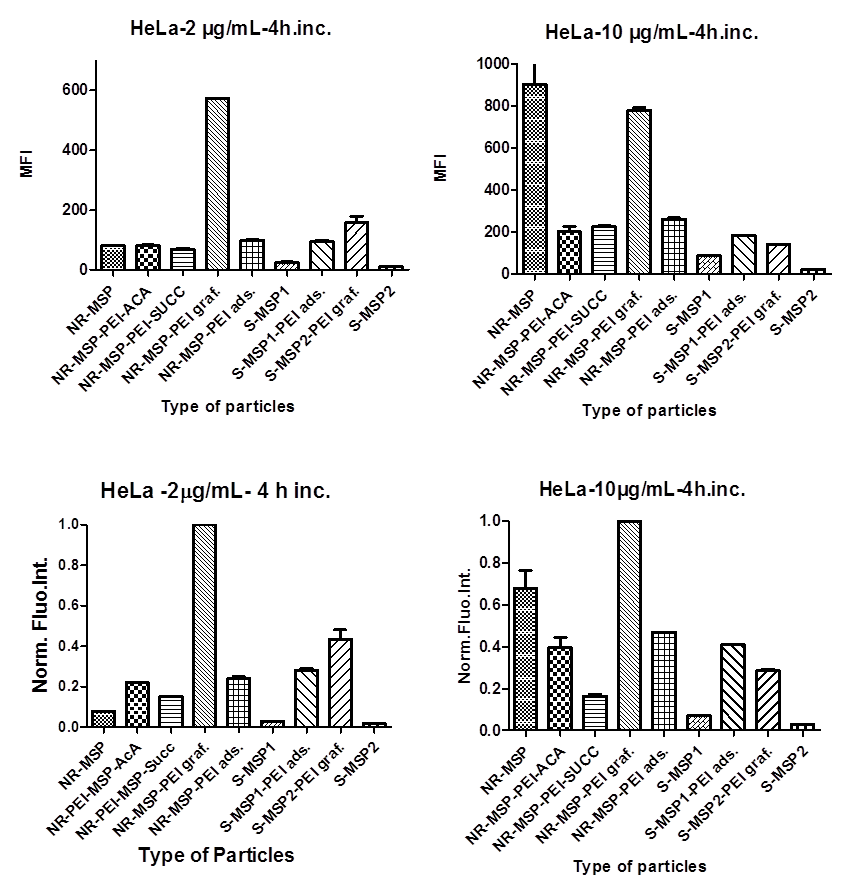

Supplement: Additional file 3 — Figure S2 MFI values from FACS for HeLa cells incubated with 10 and 2 μg/mL MSPs for 4 h. The lower graphs have been normalized against pure particle suspension fluorescence values measured at 530 nm in HEPES buffer at pH 7.2. [file 1556-276X-7-358-S3.tiff]

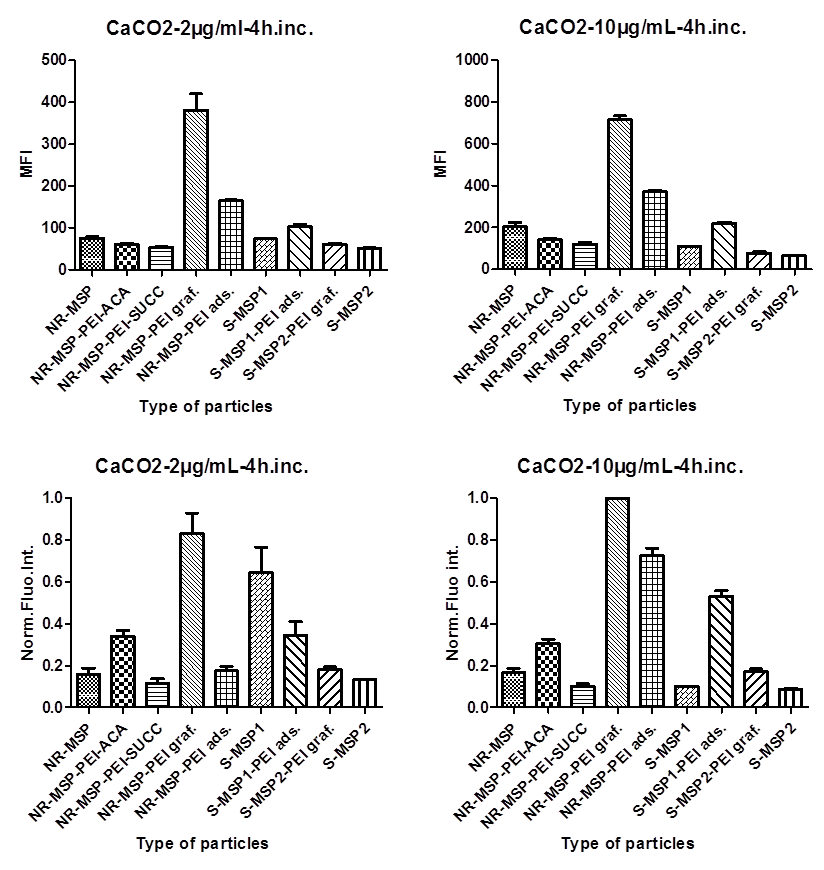

Supplement: Additional file 4 — Figure S3 MFI values from FACS for Caco-2 cells incubated with 10 and 2 μg/mL MSPs for 4 h. The lower graphs have been normalized against pure particle suspension fluorescence values measured at 530 nm in HEPES buffer at pH 7.2. [file 1556-276X-7-358-S4.tiff]

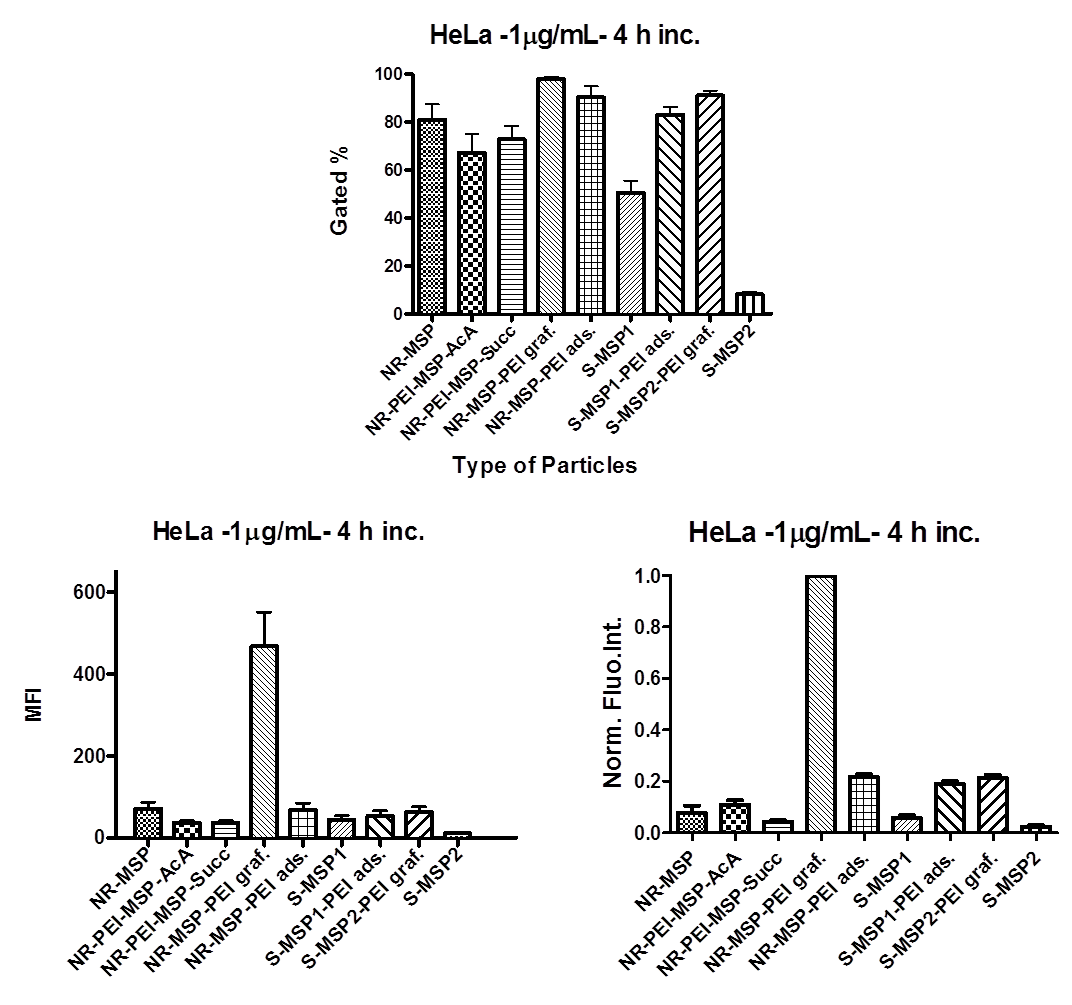

Supplement: Additional file 5 — Figure S4 MFI values from FACS for HeLa cells incubated with 1 μg/mL MSPs for 4 h. The right graph has been normalized against pure particle suspension fluorescence values measured at 530 nm in HEPES buffer at pH 7.2. [file 1556-276X-7-358-S5.tiff]
